# Withdrawal of Papers

**Published:** 2014-09-01

The paper:

VASOPRESSIN VS TERLIPRESSIN IN TREATMENT OF REFRACTORY SHOCK.

Scarpati G., Piazza O.

Transl Med UniSa. 2013 Jan 4;5:22–7. has been withdrawn after authors request.

The paper:

THE NOVEL THERAPEUTHIC TARGETS IN THE TREATMENT OF CHRONIC PAIN.

Palomba R., Bonaccia P., Graffi M., Costa F. Transl Med UniSa. 2012 Apr 30;3:57–61. has been withdrawn after authors request.

